# Analysis of Chaotic Resonance in Izhikevich Neuron Model

**DOI:** 10.1371/journal.pone.0138919

**Published:** 2015-09-30

**Authors:** Sou Nobukawa, Haruhiko Nishimura, Teruya Yamanishi, Jian-Qin Liu

**Affiliations:** 1 Department of Management Information Science, Fukui University of Technology, Fukui, Japan; 2 Graduate School of Applied Informatics, University of Hyogo, Kobe, Hyogo, Japan; Georgia State University, UNITED STATES

## Abstract

In stochastic resonance (SR), the presence of noise helps a nonlinear system amplify a weak (sub-threshold) signal. Chaotic resonance (CR) is a phenomenon similar to SR but without stochastic noise, which has been observed in neural systems. However, no study to date has investigated and compared the characteristics and performance of the signal responses of a spiking neural system in some chaotic states in CR. In this paper, we focus on the Izhikevich neuron model, which can reproduce major spike patterns that have been experimentally observed. We examine and classify the chaotic characteristics of this model by using Lyapunov exponents with a saltation matrix and Poincaré section methods in order to address the measurement challenge posed by the state-dependent jump in the resetting process. We found the existence of two distinctive states, a chaotic state involving primarily turbulent movement and an intermittent chaotic state. In order to assess the signal responses of CR in these classified states, we introduced an extended Izhikevich neuron model by considering weak periodic signals, and defined the cycle histogram of neuron spikes as well as the corresponding mutual correlation and information. Through computer simulations, we confirmed that both chaotic states in CR can sensitively respond to weak signals. Moreover, we found that the intermittent chaotic state exhibited a prompter response than the chaotic state with primarily turbulent movement.

## Introduction

By virtue of recent developments in brain measurement technology, it is now recognized that information is transmitted among neurons according to not only their firing rate but also their spike timing. Therefore, spiking neuron models, which describe spike timing, have attracted considerable attention. The Hodgkin–Huxley (HH) approach [1] is known to be the most useful spiking neuron model for simulating neurodynamics, and does so by describing the capacitance of membranes and the characteristics of the resistance of ion channels. This model is represented by four equations involving several physiological parameters pertaining to membrane potential, the activation of the Sodium (Na) and Potassium (K) ion-currents, and the inactivation of the Na ion-current. This model can reproduce almost all spiking activity observed in neural systems by tuning the parameters. However, because of the complexity of the physiological parameters involved, researchers have proposed many neuron models that use a smaller number of parameters, and hence are simpler than the HH model, by focusing on membrane potential behavior of spiking modes and response characteristics during spiking activity, such as the integrate-and-fire neuron model and the FitzHugh–Nagumo neuron model [2]. Of such simplified models, the Izhikevich neuron model [3] combines continuous spike-generation mechanisms and a discontinuous resetting process following the spikes, and can reproduce major spike patterns that have been observed experimentally by tuning a few parameters. Furthermore, the variety of the spiking properties obtained through this model is greater than those obtained through other models [4].

The Izhikevich neuron model can simulate chaotic spiking activity under certain conditions regarding specific parameter sets and given adequate numerical precision by integrating small time steps and accurately detecting the spiking points [4, 5]. Furthermore, it has been claimed that in order to determine this chaotic state, the Lyapunov exponents need to be calculated using a saltation matrix in order to account for the state-dependent jump in the resetting process [5]. Note that if the effect of the state-dependent jump is ignored, i.e., if the saltation matrix is not used, the maximum Lyapunov exponent has a positive value even in periodic states [5].

In the last few decades, researchers have realized that several processes for signal detection and transmission in neural systems are supported by the mechanism of stochastic resonance (SR) [6–8]. SR is a phenomenon whereby the presence of noise helps a nonlinear system amplify a weak (sub-threshold) signal. SR can be observed in many kinds of systems that have three ingredients: a kind of barrier/threshold, a source of noise, and a weak input signal. Chaotic processes also cause a phenomenon similar to SR called chaotic resonance (CR) with the ingredient of a deterministic fluctuating activity instead of a source of noise in stochastic processes. As regarding CR, two kinds of fluctuating activities have been considered, so far. One is the case whereby external additive chaotic signal is applied to the system instead of stochastic noise [9, 10]. The other is the case whereby external additive chaotic signal is absent and alternatively intrinsic chaotic activities are utilized. Recently, there have been many studies of CR in the latter condition. The characteristic of this CR was initially investigated using simple models [11–14] such as a one-dimensional cubic map. By focusing on the neural system, researchers observed that chaos exists at several hierarchical levels, from the electrical response of a single neuron to the activity of the entire brain as an assembly of neurons [15–18]. CR has recently been studied in neural systems, such as by using the chaotic neural network [19, 20] and the inferior olive (IO) neural system [21–24]. In [19, 20], the superior signal response capability of CR to conventional SR was exhibited by using a chaotic neural network model based on the mean firing rate of neurons. Furthermore, the work in [21–24] suggested that CR plays a part in the function that allows IO neurons to transmit error signals containing large amounts of information for cerebellar learning in continuous spiking neuron models. In the CR phenomenon in spiking neural systems, chaotic behavior leads to the generation of spikes not at specific times, but at varying scatter times for each trial as input signals. Thus, the frequency distribution of these spike timings against the input signal becomes congruent with the shape of the input signal [21–24]. However, no study to date has investigated and compared the signal responses of some chaotic states in CR revealed by the bifurcation analysis of a spiking neural system with a resetting process, such as the Izhikevich neuron model. In past research, we have analyzed the bifurcation and the signal response of CR in the Izhikevich neuron model [25–27]. However, these studies involved issues related to the accuracy of the numerical simulations [25, 26] and the quantitative bifurcation analysis [27].

In this paper, we examine and classify the chaotic characteristics of the Izhikevich neuron model by using Lyapunov exponents with a saltation matrix and Poincaré section methods in order to address the measurement problem caused by the state-dependent jump in the resetting process. Following the structure adopted in our past work [25–27], we then rigorously evaluate the signal response in CR for classified chaotic states through a numerical verification method [28] and a quantitative method to specify the bifurcation in the system with the resetting process [29].

## Izhikevich neuron model

The Izhikevich neuron model [3, 4] is a two-dimensional (2D) system of ordinary differential equations of the form

$$\begin{array}{c} v^{\prime }=0.04v^{2}+5v+140-u+I, \end{array}$$ (1)

$$u^{\prime }=a\left(bv-u\right).$$ (2)

**Fig 1:**
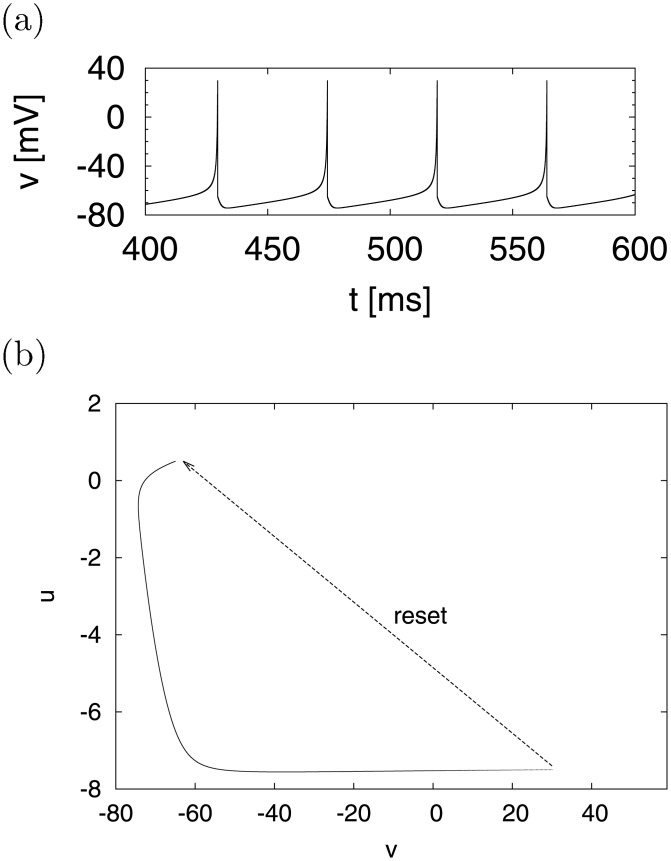
System behavior in case of regular spiking (RS). (a) Time evolution of *v*(*t*). (b) Typical trajectory, including state-dependent jump, in the (*v*, *u*) phase plane (*a* = 0.02, *b* = 0.2, *c* = −65, *d* = 8, *I* = 10 [3]).

In the above equation, *v* and *u* represent the membrane potential of a neuron and the membrane recovery variable, respectively. *v* and time *t* are measured in [mV] and [ms], respectively. When the membrane potential *v* > 30 [mV], the model fires; *v* is set to *c*, and *u* is set to *u* + *d*, which is called the resetting process. *I* is the direct current (DC) input. The parameters *a* and *b* describe the time scale and the sensitivity of *u*, respectively. Spiking behavior, such as regular spiking, intrinsically bursting, and fast spiking can be reproduced using this model. As an example of regular spiking (*a* = 0.02, *b* = 0.2, *c* = −65, *d* = 8, *I* = 10) [3], Fig 1 (a) and (b) show the time evolution of *v*(*t*) and the system trajectory in a phase plane (*v*, *u*), respectively. Due to the resetting process, when *v*(*t*) exceeds 30 [mV], the system state (*v*, *u*) (discontinuously) jumps to point ((*v*, *u*) ≈ (−65, 0.3)), as shown in Fig 1 (b). In our simulation, we numerically analyzed this model through non-linear differential/algebraic equation solvers by using the backward differentiation formula method [28] to achieve sufficient numerical precision in order to evaluate chaotic spiking activity. This method is more precise than Euler’s method, which was adopted to reproduce only periodic spiking in [3].

To determine the uniformity of the neuron spikes, we adopt the coefficient of variation for inter-spike intervals [30]:

$$CV=\frac{\sqrt{Var(T_{k})}}{<T_{k}>}.$$ (3)

*T*_*k*_ is the *k*-th spike interval (*T*_*k*_ = *t*_*k* + 1_ − *t*_*k*_), and *Var*(*T*_*k*_) and < *T*_*k*_ > are the variance and the mean of *T*_*k*_, respectively. *CV* here becomes 0 in the one-period state and positive in the non-periodic state, including chaotic states.

As an index to check whether a state is chaotic, the maximum Lyapunov exponent is ordinarily used for systems with continuous trajectories, and is calculated by

$$\lambda_{1}=\frac{1}{\tau N}\sum_{k=1}^{N}\ln\left(\frac{|d^{k}\left(t_{l}=\tau \right)|}{|d^{k}\left(t_{l}=0\right)|}\right).$$ (4)

Here, ∣**d** ^*k*^(*t*_*l*_ = 0)∣ (*k* = 1, 2, ⋯, *N*) are *N* perturbed initial conditions applied to the system trajectory at *t*_*l*_ = 0, and **d** ^*k*^(*t*_*l*_ = *τ*) represent their evolution in time for *t*_*l*_ ∈ [0 : *τ*] [31]. Let us consider the case where a neuron fires at *t*_*l*_ = *t*_*s*_, *k* = *i*. Since the time evolution of the system’s trajectory is discontinuous in the resetting process, **d** ^*i*^(*t*_*s*_) receives the interruption, and ∣**d** ^*i*^(*τ*)∣ is rendered irrelevant to the evolution of the system’s trajectory. Due to this influence, *λ*_1_ loses its accuracy in such situations. Therefore, trials for new measures of such a system are needed [29, 32, 33]. One such proposed measure is the insertion of the Lyapunov exponent into a saltation matrix in order to address the stability of the system’s trajectory, including state-dependent jumps [5]. Therefore, we use the Lyapunov exponent with a saltation matrix to analyze chaotic states in the Izhikevich neuron model.

## Fundamental properties of the model

**Fig 2:**
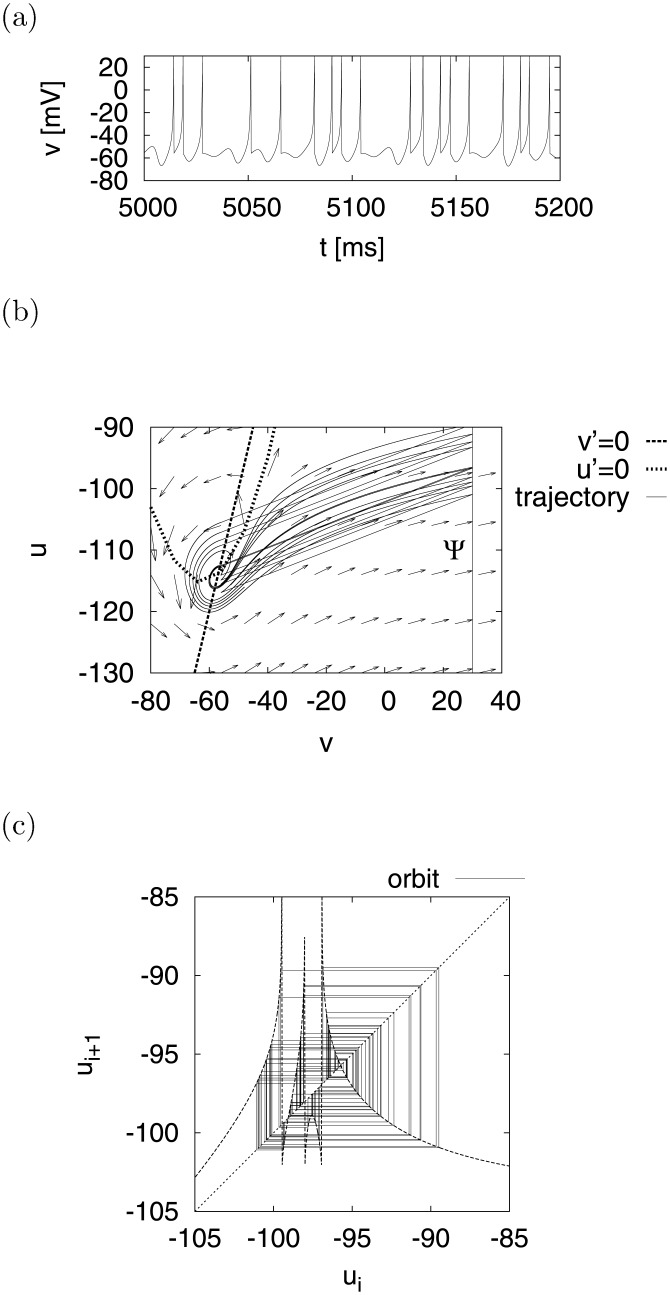
Chaotic system behavior for *d* = −16. (a) Time evolution of *v*(*t*). (b) Its trajectory in the (*v*, *u*) phase plane. The dashed line represents the *v*-nullcline (*v*′ = 0) and the dotted line represents the *u*-nullcline (*u*′ = 0). The arrows indicate the vector field of *v* and *u*. (c) The return map of (*u*_*i*_, *u*_*i* + 1_), where the solid line represents the orbit of *u*_*i*_, the dotted line represents the solution of *u*_*i* + 1_ = *ψ*(*u*_*i*_), and the dashed line depicts *u*_*i* + 1_ = *u*_*i*_. (*a* = 0.2, *b* = 2, *c* = −56, *I* = −99, *d* = −16).

The Izhikevich neuron model can reproduce major firing patterns, such as regular spiking, intrinsically bursting, chattering, and fast spiking [3, 4]. Moreover, research has suggested that this model can simulate chaotic behavior with appropriate parameter values (*a* = 0.2, *b* = 2, *c* = −56, *d* = −16, *I* = −99 in Eqs (1) and (2) [4]. Fig 2 (a) and (b) show the chaotic time evolution of *v*(*t*) and the strange attractor in a phase plane (*v*, *u*), respectively. We also examine the strange attractor in greater detail by using the Poincaré sections Ψ (*v* = 30 [mV]). The dynamics of (*u*_1_, *u*_2_,⋯, *u*_*N*_), which is the evolution of *u* over time on Ψ, is defined as a Poincaré mapping *u*_*i* + 1_ = *ψ*(*u*_*i*_). As shown in Fig 2(c), on the return map (*u*_*i*_, *u*_*i* + 1_), the solution for *u*_*i* + 1_ = *ψ*(*u*_*i*_), the orbit of *u*_*i*_ and *u*_*i* + 1_ = *u*_*i*_ are indicated by dotted, solid, and dashed lines, respectively. It has been observed that the orbit of *u*_*i*_ exhibits chaotic behavior in the range −102 ≲ *u*_*i*_ ≲ −90, and the shape of *ψ* displays a stretching and folding structure as a feature of the non-linear map.

To quantify this chaotic activity in the Izhikevich neuron model, we use the Lyapunov exponent with a saltation matrix. On a system with a continuous trajectory between the *i*-th and the (*i* + 1)-th spiking times, (*t*_*i*_ ≤ *t* ≤ *t*_*i* + 1_), the variational Eqs (1) and (2) are defined as follows:

$$\begin{array}{c} {\dot{\Phi }}_{i+1}\left(t,t_{i}\right)=J\left(v,u,t\right)\Phi_{i+1}\left(t,t_{i}\right), \end{array}$$ (5)

$$\Phi_{i+1}\left(t_{i},t_{i}\right)=E,$$ (6)

where, Φ, *J*, and *E* indicate the state transition matrix, the Jacobian matrix, and a unit matrix, respectively. At *t* = *t*_*i*_, the saltation matrix is given by

$$\begin{array}{c} S_{i}=[\begin{array}{cc} \frac{{\dot{v}}^{+}}{{\dot{v}}^{-}} & 0\\ \frac{{\dot{u}}^{+}-{\dot{u}}^{-}}{{\dot{v}}^{-}} & 1 \end{array}]. \end{array}$$ (7)

In the above, (*v* ^−^, *u* ^−^) and (*v* ^+^, *u* ^+^) represent the values of (*v*, *u*) before and after spiking, respectively. In case spikes arise in the range [*T* ^*k*^:*T* ^*k* + 1^] [ms], Φ^*k*^(*T* ^*k* + 1^, *T* ^*k*^) (*k* = 0, 1, ⋯, *N* − 1) [5] can be expressed as

$$\Phi^{k}\left(T^{k+1},T^{k}\right)=\Phi_{i+1}\left(T^{k+1},t_{i}\right)S_{i}\Phi_{i}\left(t_{i},t_{i-1}\right)\cdots S_{2}\Phi_{2}\left(t_{2},t_{1}\right)S_{1}\Phi_{1}\left(t_{1},T^{k}\right).$$ (8)

By using the eigenvalues $l_{j}^{k}\left(j=1,2\right)$ of Φ^*k*^(*T* ^*k* + 1^, *T* ^*k*^), the Lyapunov spectrum *λ*_*j*_ is calculated by

$$\lambda_{j}=\frac{1}{T^{N}-T^{0}}\sum_{k=0}^{N-1}\log(|l_{j}^{k}\left|\right).$$ (9)

In our simulation, we set *T* ^*k* + 1^ − *T* ^*k*^ as the time required for 20 spikes (*i* = 20). We set 1000 [ms] as the maximum value in case *T* ^*k* + 1^ − *T* ^*k*^ takes 1000 [ms] before 20 spikes occur.

**Fig 3:**
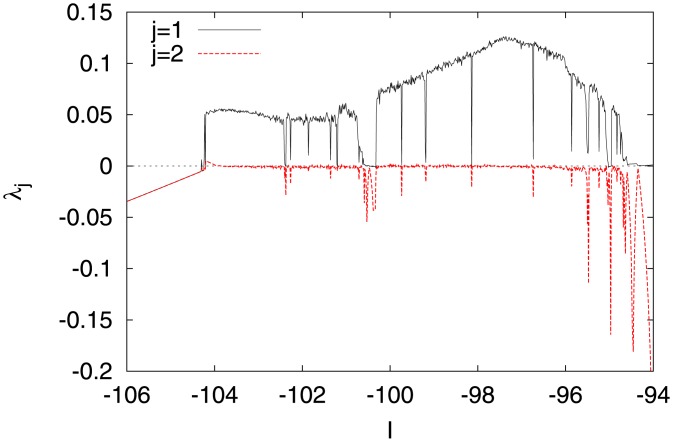
Dependence of Lyapunov exponents *λ*_*j*_ (*j* = 1, 2) on the input DC current *I* (*a* = 0.2, *b* = 2, *c* = −56, *d* = −16).

We investigated the behavior of the system in detail by enlarging the parameter region on *I* and *d*, including the values *I* = −99 and *d* = −16 used in Fig 2. Fig 3 shows the dependence of *λ*_*j*_ on *I*, obtained under the condition that the values of the other parameters were fixed to those shown in Fig 2. Chaotic behavior was observed (*λ*_1_ > 0) within a certain range on either side of *I* = −99 (−104.5 ≲ *I* ≲ −94.5). Furthermore, the system came to rest (non-firing) (*λ*_1_ < 0, *λ*_2_ < 0) for *I*≲ −104.5, whereas periodic firing (*λ*_1_ ≈ 0, *λ*_2_ < 0) was observed at *I* ≳ −94.5.

**Fig 4:**
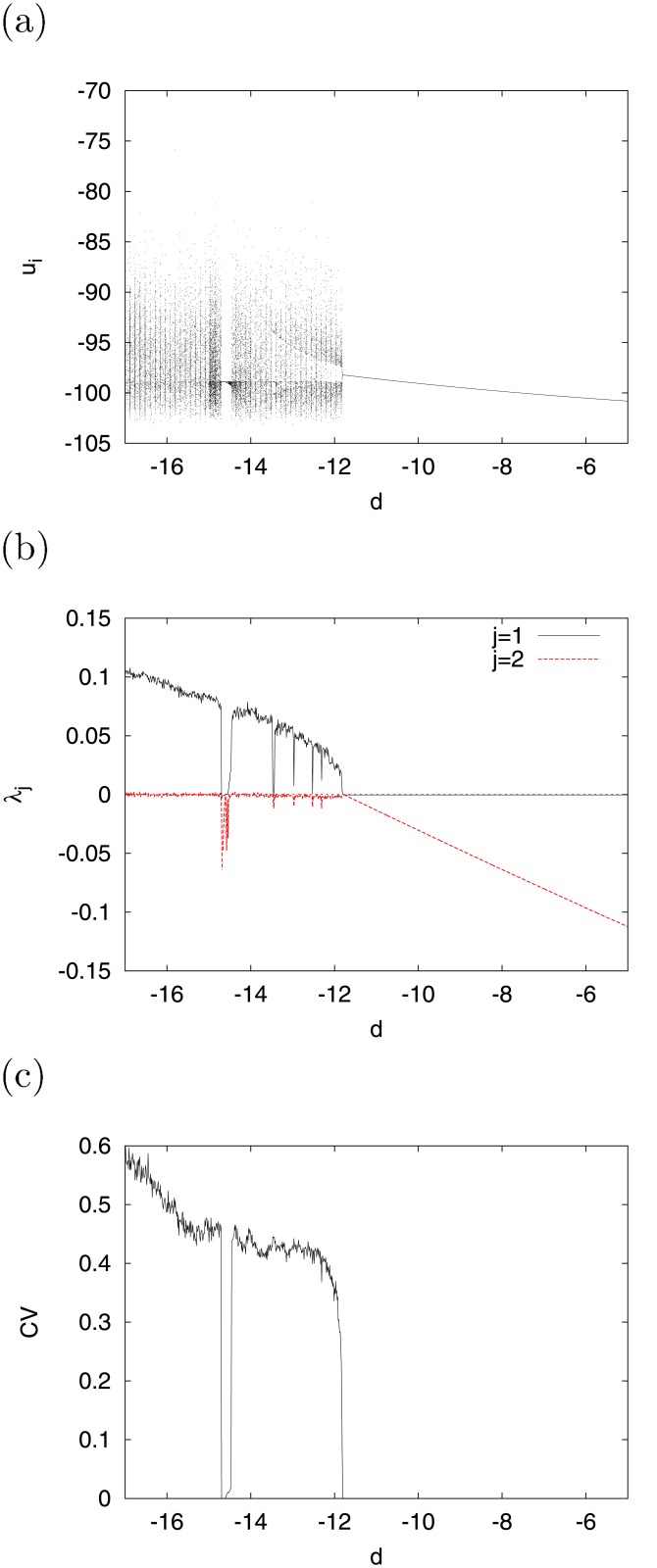
Dependence of bifurcation on parameter *d*. (a) Bifurcation diagram of *u*_*i*_. (b) Lyapunov exponents *λ*_*j*_ (*j* = 1, 2). (c) Coefficient of variation for inter-spike interval *CV* (*a* = 0.2, *b* = 2, *c* = −56, *I* = −99).

By fixing the value of *I* at −99, we investigated the bifurcation of this system by replacing *I* with *d* by using a bifurcation diagram consisting of (*u*_1_, *u*_2_, ⋯ , *u*_*N*_). Fig 4(a), (b), and (c) represent the bifurcation diagrams of *u*_*i*_, *λ*_*j*_, and *CV*, respectively, as functions of *d*. For *d* ≲ −11.9, the chaotic trajectory was distributed in the range −103 ≲ *u*_*i*_ ≲ −80, and the system exhibited a chaotic state (*λ*_1_ > 0) and irregular spiking activity (*CV* > 0), excluding periodic windows (*λ*_1_ = 0), given periodic-1 (*CV* = 0) and multiple periodic (*CV* > 0) states. As the value of *d* increased, those of *λ*_1_ and *CV* decreased. They converged at 0 for *d* ≳ −11.9, i.e., the system assumed periodic states and exhibited periodic spiking. In order to conduct bifurcation analysis in the system with a state-dependent jump, we used the evaluation method intended to assess the stability of a fixed point *u*_0_ = *ψ* ^*l*^(*u*_0_) (*l* = 1, 2, ⋯) on a Poincaré section. In the literature [29], this stability has been evaluated by

$$\mu =\frac{\partial \phi^{l}}{\partial u_{0}}=(\begin{array}{cc} 0 & 1 \end{array})(\begin{array}{cc} 0 & 0\\ -\dot{v}/\dot{u} & 1 \end{array})\Phi \left(t_{l},t_{0}\right)(\begin{array}{c} 0\\ 1 \end{array}).$$ (10)

In the above, **u**_0_ = (*v*_0_, *u*_0_) indicates the initial value of orbit **u** = (*v*, *u*) at *t* = *t*_0_. ∣*μ* < 1∣, *μ* = −1, and *μ* = 1 represent the stable condition, period doubling bifurcation, and tangent bifurcation, respectively. In Fig 4, the tangent bifurcation at *l* = 2 arises at *d* ≈ −11.9. Through this tangent bifurcation, the system transitions into chaos at *d* ≲ −11.9, as shown in Fig 4(a) and (b).

**Fig 5:**
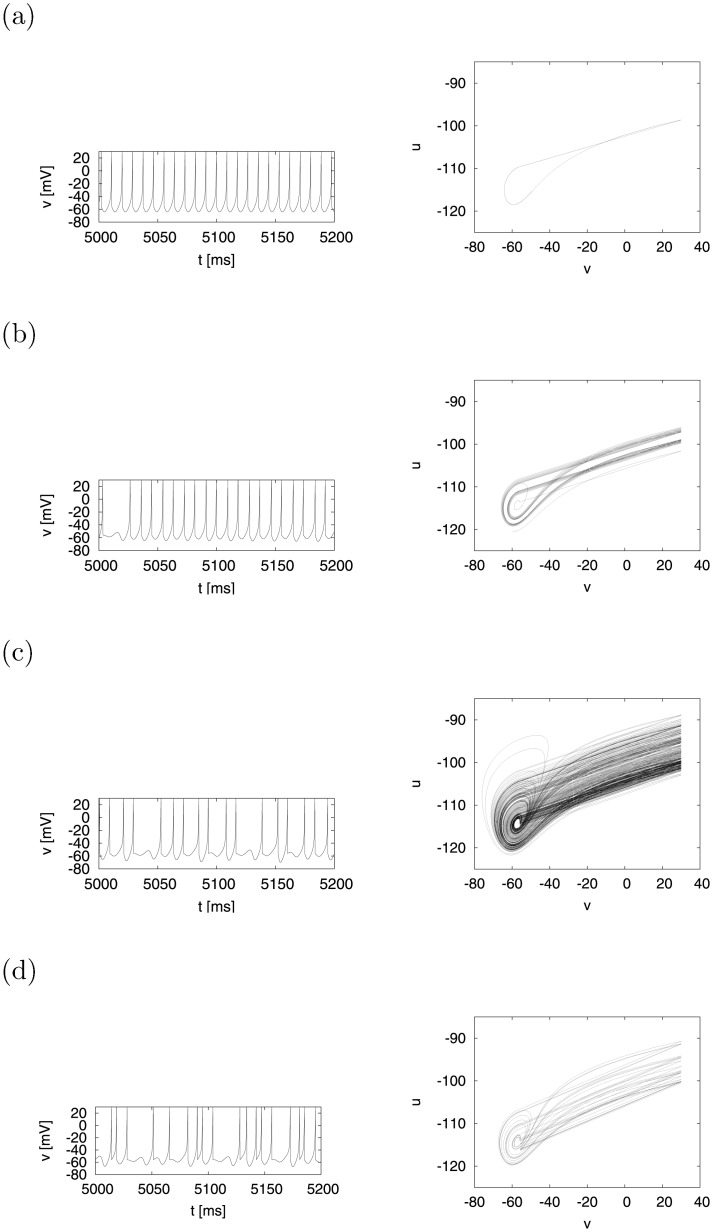
Time series of membrane potential *v*(*t*) (left) and attractor (right). (a) *d* = −11, (b) *d* = −12, (c) *d* = −13, (d) *d* = −16 (*a* = 0.2, *b* = 2, *c* = −56, *I* = −99).

Furthermore, in the region *d* ≈ −11.9 as a bifurcation point, the trajectory (*v*, *u*) and the time series of *v*(*t*) were examined. At *d* = −11 in Fig 5(a), the time series of *v*(*t*) (left) and the trajectory (right) indicate periodic spiking and a one-period state, respectively. As the value of *d* decreases, the behavior of the system becomes irregular, as shown in Fig 5(b), (c), and (d). It can be observed that the durations of the apparently periodic instances of spiking seemed to have decreased during episodes of chaotic behavior in the system with a reduction in the value of *d*.

**Fig 6:**
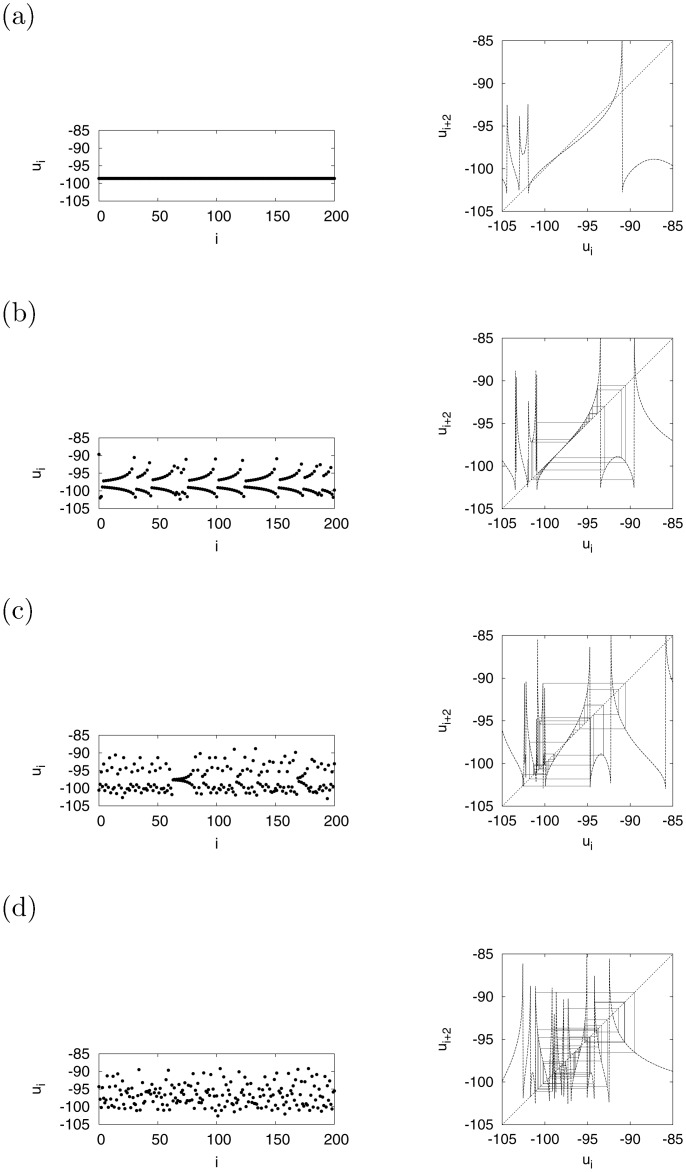
System behavior at the Poincaré section. Time series of *u*_*i*_ (left). Return map of (*u*_*i*_, *u*_*i* + 2_) (right). The solid line represents the orbit of *u*_*i*_, the dotted line shows the solution to *u*_*i* + 2_ = *ψ* ^2^(*u*_*i*_), and the dashed line depicts *u*_*i* + 2_ = *u*_*i*_. (a) *d* = −11, (b) *d* = −12, (c) *d* = −13, (d) *d* = −16 (*a* = 0.2, *b* = 2, *c* = −56, *I* = −99).

Following this, we evaluated in greater detail the behavior of the system shown in Fig 5 by using the Poincaré section method. Fig 6 (left) shows the time series of *u*_*i*_ as system behavior on the Poincaré section Ψ. As shown in Fig 6(a) (left), the value of *u*_*i*_ remained fixed (*u*_*i*_ ≈ −98.6). At *d* = −12 (Fig 6(b) (left)), the value of *u*_*i*_ began to oscillate with a focus on *u*_*i*_ ≈ −98.6. This oscillation expanded to −102 ≲ *u*_*i*_ ≲ −90, following which the value of *u*_*i*_ reverted to approximately −98.6. The periodic oscillation disappeared gradually as the value of *d* decreased, as shown in Fig 6(b) (*d* = −12), (c) (*d* = −13), and (d) (*d* = −16). In order to focus on the oscillatory behavior of *u*_*i*_, we used the return map (*u*_*i*_, *u*_*i* + 2_). Fig 6 (right) shows the orbit of *u*_*i*_ (solid line), and the solutions to *u*_*i* + 2_ = *ψ* ^2^(*u*_*i*_) (dotted line) and *u*_*i* + 2_ = *u*_*i*_ (dashed line). When *d* = −11 ((a)), the orbit of *u*_*i*_ remained at the intersection (≈ (−98.5,−98.5)) of *u*_*i* + 2_ = *ψ* ^2^(*u*_*i*_) and *u*_*i* + 2_ = *u*_*i*_, and there were two unstable fixed points on both sides of this stable fixed point at *u*_*i*_ ≈ −101.5 and −91.5. As noted above, the tangent bifurcation arose at *d* ≈ −11.9, i.e., a pair consisting of an unstable fixed point and a stable one destroyed each other. Through this tangent bifurcation, at *d* = −12 ((b)), the orbit of *u*_*i*_ exhibited sluggish movement (called laminar state) in the region where the slope of *ψ* ^2^ was approximately 1.0 (−102 ≲ *u*_*i*_ ≲ −94), and irregularly active movement (called turbulent or burst state) in regions with larger slopes (≫ 1). Such chaotic, dynamic alternation between laminar and turbulent states is called intermittency chaos [34, 35]. Note that the term of burst is not used in this paper to avoid confusion between the chaotic movement and the neural spike patterns in neurodynamics such as intrinsically bursting and chattering bursting. As the value of *d* decreased, the area of the region producing the laminar state, where the slope of *ψ* ^2^ was approximately 1.0, shrunk as well. The turbulent state was subsequently dominant in the dynamics, i.e., the system transitioned from intermittency chaos to chaos involving primarily turbulent movement, as shown in Fig 6 (b)–(d).

We thus confirmed that in the parameter region (*a* = 0.2, *b* = 2, *c* = −56, *d* = −16, *I* = −99) proposed by Izhikevich as the parameter set that produces chaotic behavior, the periodic state transitions into chaos through tangent bifurcation and intermittency chaos, i.e., the intermittent route to chaos exists in this region.

## Response efficiency in chaotic resonance

In this section, in order to reveal the efficiency of signal response in a chaotic state, we investigated the response of the system to a weak periodic signal that could not be detected in a periodic state. First, we introduced an extended Izhikevich neuron model with a signal. Second, to measure the efficiency of the response to the weak periodic signal, we defined the evaluation indices of mutual correlation and information. Third, we evaluated the dependence of the efficiency of signal response on parameter *d*, signal strength *A* and signal frequency *f*_0_ by using these evaluation indices.

### Extended Izhikevich neuron model with a periodic signal

We extended Eqs (1) and (2) using a weak periodic signal *S*(*t*) as follows:

$$\begin{array}{c} v^{\prime }=0.04v^{2}+5v+140-u+I+S\left(t\right), \end{array}$$ (11)

$$u^{\prime }=a\left(bv-u\right),$$ (12)

where we adopted *S*(*t*) = *A*sin(2*πf*_0_ *t*). It is noteworthy that the sinusoidal signal was utilized merely as a typical example of a signal in a neural system. As with Eqs (1) and (2), if the membrane potential *v* > 30 [mV], *v* was set to *c* and *u* was set to *u* + *d*. In the following simulation, we set the parameter values to (*a*, *b*, *c*, *I*) = (0.2, 2, −56, −99) in addition to values shown in Fig 4 and *f*_0_ = 0.1.

We calculated the timing of the spikes against signal *S*(*t*) by using a cycle histogram $F(\tilde{t})$. $F(\tilde{t})$ was a histogram of firing counts at *t*_*k*_ mod (*T*_0_) (*k* = 1, 2, ⋯) against signal $S(\tilde{t})$ with period *T*_0_(= 1/*f*_0_), $-T_{0}/2\leq \tilde{t}\leq T_{0}/2$. For example, for *T*_0_ = 10, in case the spike times were *t*_*k*_ = 2, 6, 12, 16, 26, the values of *t*_*k*_ mod (*T*_0_) were 2, 6, 2, 6, 6, which corresponded to $\tilde{t}=2,-4,2-4,-4$ in ${\tilde{t}}_{k}$. The cycle histogram then became *F*(2) = 2 and *F*(−4) = 3.

To quantify the signal response, we used the following indices, i.e., Eqs (13) and (14) and Eqs (15)–(17). The mutual correlation *C*(*τ*) between the cycle histogram $F(\tilde{t})$ of the neuron spikes and the signal $S(\tilde{t})$ is given by

$$\begin{array}{c} C\left(\tau \right)=\frac{C_{SF}\left(\tau \right)}{\sqrt{C_{SS}C_{FF}}}, \end{array}$$ (13)

$$C_{SF}\left(\tau \right)=<\left(S\left(\tilde{t}+\tau \right)-<S\left(\tilde{t}\right)>\right)\left(F\left(\tilde{t}\right)-<F\left(\tilde{t}\right)>\right)>.$$ (14)

For the time delay factor *τ*, we checked max_*τ*_ *C*(*τ*), i.e., the largest *C*(*τ*) between $\frac{-T_{0}}{2}\leq \tau \leq \frac{T_{0}}{2}$.

As an extensively used index for evaluating information transmission, we used mutual information, which is information transmitted from input *S* to output *F*,

MI(F;S)=H(F)-H(F|S). (15)

Here, *S* and *F* consisted of *m*_*s*_ (*s*_1_, *s*_2_, ⋯, *s*_*m*_*s*__) and *m*_*f*_ (*f*_1_, *f*_2_, ⋯, *f*_*m*_*f*__) event states, into which $S(\tilde{t})$ (−*A* ∼ *A*) and $F(\tilde{t})$ (0 ∼ its maximum value) were equally divided, respectively. *H*(*F*) and *H*(*F*∣*S*) are given by

$$H\left(F\right)=-\sum_{j}P\left(f_{j}\right)\log_{2}P\left(f_{j}\right),$$ (16)

$$H\left(F|S\right)=-\sum_{i}\sum_{j}P\left(s_{i}\right)P\left(f_{j}|s_{i}\right)\log_{2}P\left(f_{j}|s_{i}\right),$$ (17)

where *P*(*s*_*j*_) and *P*(*f*_*j*_) represent the occurrence probability of *s*_*j*_ and *f*_*j*_, and *P*(*f*_*j*_∣*s*_*i*_) is the conditional probability for the occurrence of *f*_*j*_ and *s*_*i*_. In our simulation, we set *m*_*s*_ = *m*_*f*_ = 20. However, if the maximum value of $F(\tilde{t})$ was smaller than that of *m*_*f*_, *m*_*f*_ was set to the maximum value because $F(\tilde{t})$ was the integer of firing counts.

### Dependence on parameter *d*

**Fig 7:**
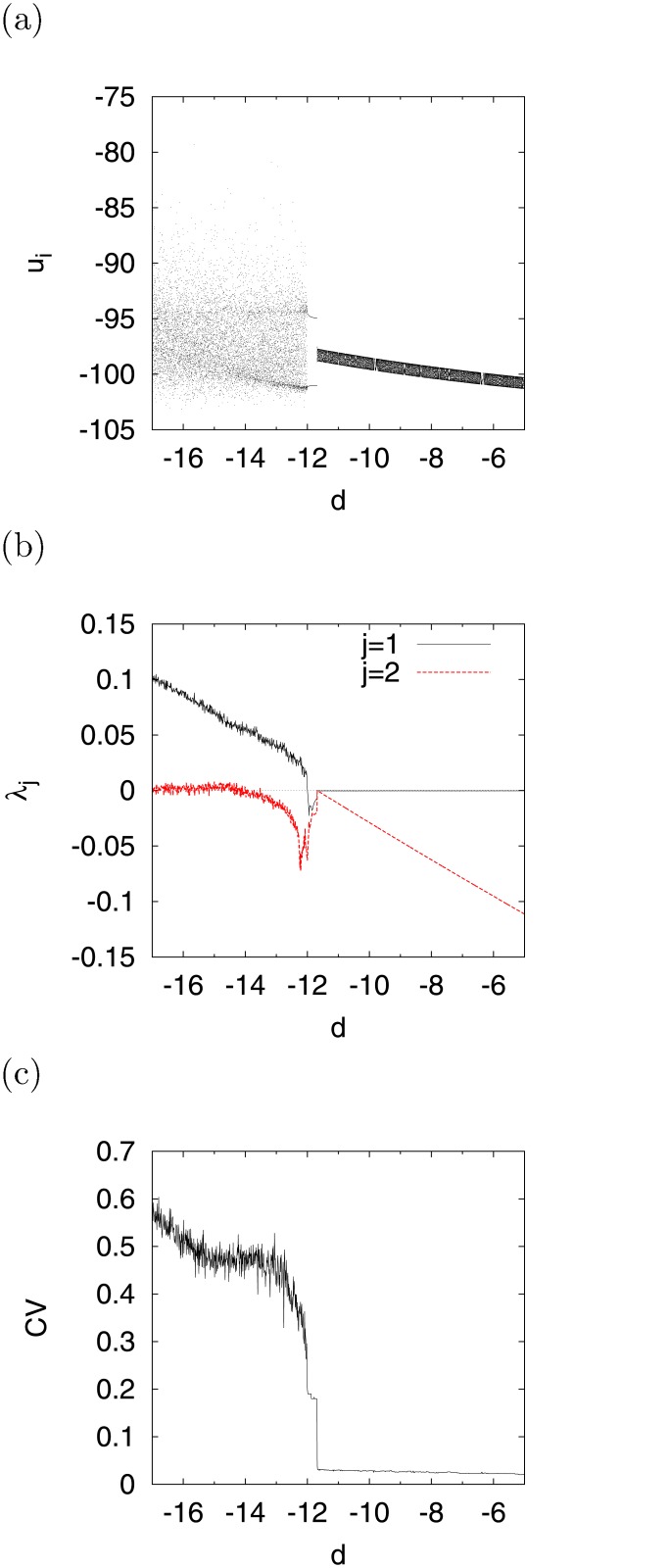
Dependence of bifurcation on parameter *d* under weak sinusoidal signal. (a) Bifurcation diagram of *u*_*i*_.(b) Lyapunov exponents *λ*_*j*_ (*j* = 1, 2). (c) Coefficient of variation for inter-spike interval *CV*. (*a* = 0.2, *b* = 2, *c* = −56, *I* = −99, *A* = 0.3, *f*_0_ = 0.1).

This section concerns the response of the system represented by Eq (11). As mentioned in the section “Fundamental properties of the model,” the system featured periodic firing activity in the region (−12 ≲ *d* ≲ −5) and chaotic activity, which was generated through the intermittent route to chaos, in the region (−17 ≲ *d* ≲ −12). We now examine the behavior of the system in the Izhikevich neuron model with a weak sinusoidal signal *S*(*t*) (*A* = 0.3). Fig 7 shows the bifurcation diagram of *u*_*i*_ ((a)), *λ*_*j*_ ((b)), and *CV* ((c)) as a function of *d*. In the region −17 ≲ *d* ≲ −12, the behavior of *u*_*i*_ exhibited chaotic activity (*λ*_1_ > 0). However, as *d* increased, *λ*_1_ and *CV* decreases; the system was entrained by *S*(*t*) (*λ*_1, 2_ < 0), and exhibited a period-2 state at −12 ≲ *d* ≲ −11.5. In the region −11.5 ≲ *d* ≲ −5, the system exhibited a periodic state (*λ*_1_ ≈ 0, *λ*_2_ < 0) and *u*_*i*_ showed a slight motion when the range of *u*_*i*_ was ≈ 1.

**Fig 8:**
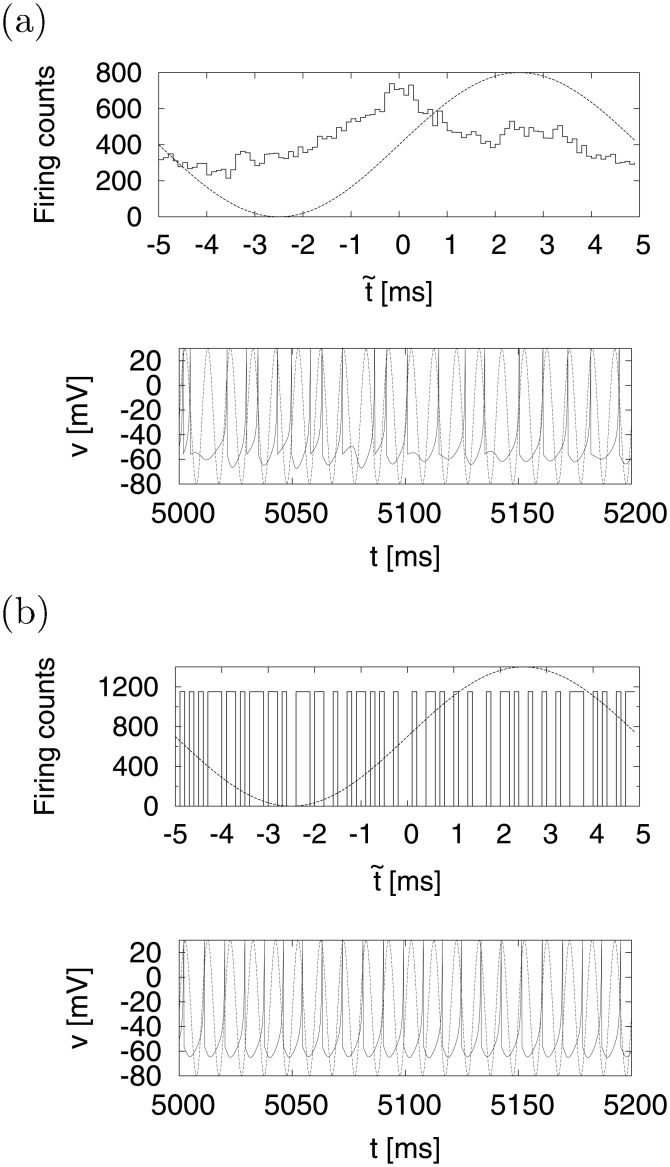
Cycle histogram $F(\tilde{t})$ (top) and time series of *v*(*t*) (bottom). In cases of (a) chaotic firing (*d* = −16) and (b) periodic firing (*d* = −10). $F(\tilde{t})$ is a histogram of firing counts at *t*_*k*_ mod (*T*_0_) (*k* = 1, 2, ⋯). The dotted lines are the input signals $S(\tilde{t})$ (*a* = 0.2, *b* = 2, *c* = −56, *I* = −99, *A* = 0.3, *f*_0_ = 1/*T*_0_ = 0.1).

**Fig 9:**
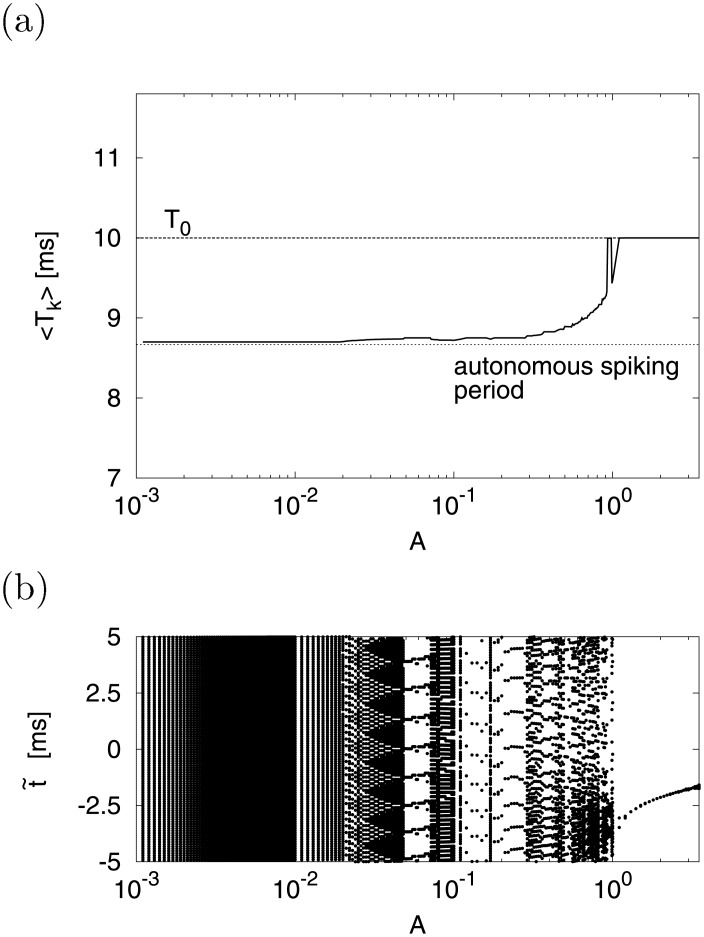
Dependence of spike timing on signal strength *A* in periodic state. (a) Mean of inter spike interval < *T*_*k*_ >. (b) Spike timing $\tilde{t}$ against input signal. (*a* = 0.2, *b* = 2, *c* = −56, *d* = −10, *I* = −99, *f*_0_ = 0.1).

Fig 8 shows the time series of *v*(*t*) (bottom) and the corresponding cycle histogram $F(\tilde{t})$ of the firing counts (top). In case *d* = −16 ((a)), the neuron fired non-periodically and the cycle histogram $F(\tilde{t})$ responded to the signal $S(\tilde{t})$ with some delay ∣*τ*∣ ≈ 3[ms]. However, when we changed the value of *d* to the periodic region, as shown in *d* = −10 ((b)), the neuron fired periodically and the cycle histogram $F(\tilde{t})$ did not respond to signal $S(\tilde{t})$. Added to this, we investigated the signal response in the periodic region in detail. Fig 9 indicates < *T*_*k*_ > ((a)) and distribution of $\tilde{t}$ ((b)) as a function of *A* in case *d* = −10. In 1 × 10^−3^ ≲ *A* ≲ 2 × 10^−2^, < *T*_*k*_ > kept about 8.7 [ms] as a period of autonomous spiking (dashed line) and $\tilde{t}$ spread over entire area (−5 to 5 [ms]) uniformly. However, < *T*_*k*_ > began to converge to 10(= *T*_0_) [ms] with increasing *A* in 2 × 10^−2^ ≲ *A* ≲ 1. In this region, $\tilde{t}$ tended to gather at the specific points by the interaction effect of *S*(*t*). Here, the signal amplitude (*A* = 0.3) used in Fig 8 belonged to this region. In cases of higher signal strength (1 ≲ *A* ≲ 3), < *T*_*k*_ > and $\tilde{t}$ were locked at 10(= *T*_0_) [ms] and some specific point $\tilde{t_{0}}$ within −3.5 ≲ $\tilde{t_{0}}$ ≲ −1.5 [ms], respectively.

**Fig 10:**
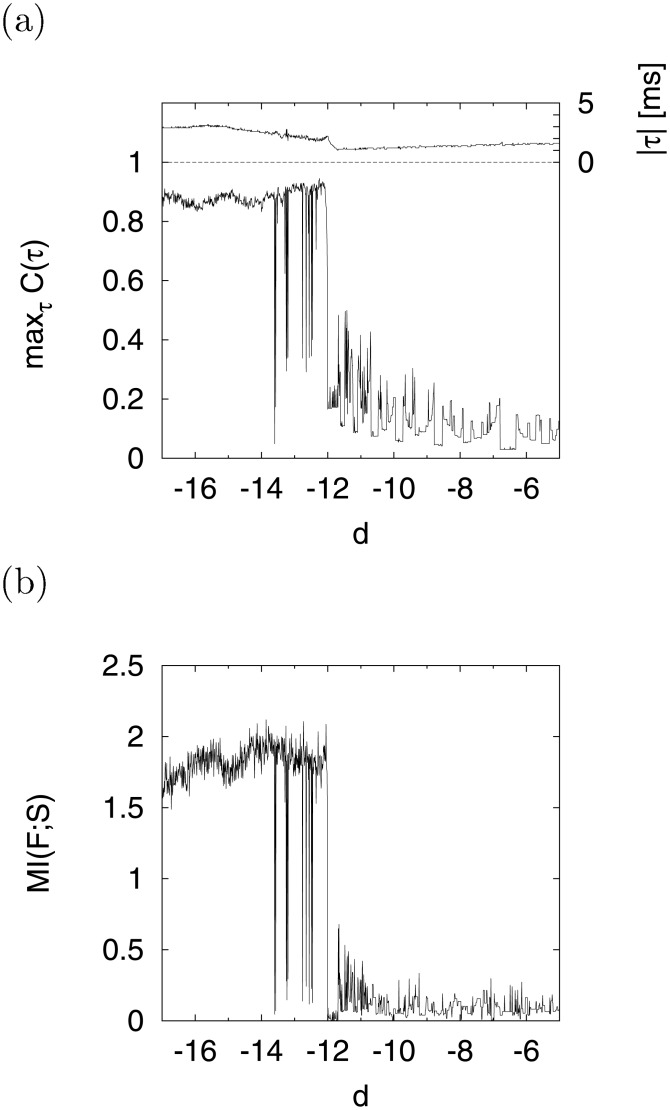
Dependence of signal response on parameter *d* in CR. (a) *d* dependence of max_*τ*_ *C*(*τ*) between cycle histogram $F(\tilde{t})$ and input signal $S(\tilde{t})$. The upper part of this figure shows the time delay ∣*τ*∣, i.e., these values realize the maximum value of *C*(*τ*). (b) *d* dependence of *MI*(*F*; *S*) between cycle histogram $F(\tilde{t})$ and input signal $S(\tilde{t})$. (*a* = 0.2, *b* = 2, *c* = −56, *I* = −99, *A* = 0.3, *f*_0_ = 0.1).

Furthermore, we evaluated max_*τ*_ *C*(*τ*) and *MI*(*F*;*S*) for the system shown in Fig 10 (a) and (b), respectively. Here, the upper part of the figure (a) shows the value of ∣*τ*∣ required to realize the maximum value of *C*(*τ*). In the region −17 ≲ *d* ≲ −13, where the system exhibited chaotic activity, the value of max_*τ*_ *C*(*τ*) increased (≈ 0.9) with the time delay ∣*τ*∣ ≈ 3 [ms]. Thus, chaotic resonance (CR) arose in this region. As with max_*τ*_ *C*(*τ*), *MI*(*F*; *S*) also maintained a high value (≳ 1.8) in the region (−17 ≲ *d* ≲ −13), as shown in Fig 10 (b).

### Sensitivity of signal response in chaotic resonance

**Fig 11:**
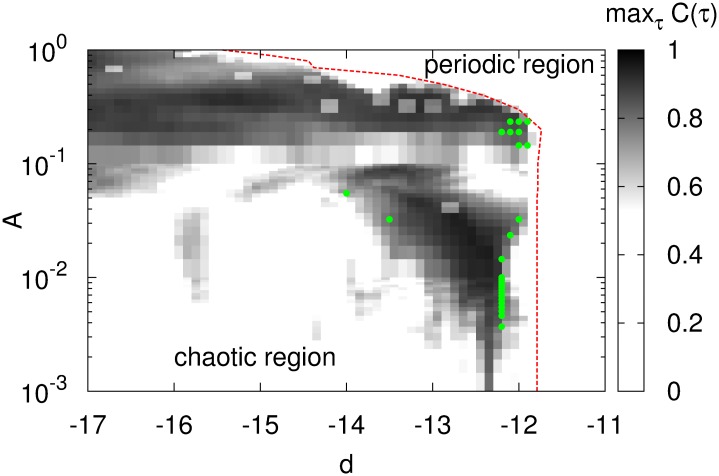
Dependence of max_*τ*_ *C*(*τ*) on parameter *d* and signal strength *A*. The dotted red line represents the *d*-threshold of *λ*_1_ > 0 (*d*_thr_) at each value of signal strength *A* (*a* = 0.2, *b* = 2, *c* = −56, *I* = −99, *f*_0_ = 0.1).

In the section “Fundamental properties of the model,” we observed that the chaotic state with primarily turbulent movement and the intermittent chaotic state coexisted in the region −17 ≲ *d* ≲ −12. In this section, we examine the sensitivity of signal response in the region of parameter *d*, including in the two chaotic states. Fig 11 shows the dependence of max_*τ*_ *C*(*τ*) on parameter *d*, as well as the signal strength *A* and the *d*-threshold of *λ*_1_ > 0 (*d*_thr_; indicated by the dotted red line) at each value of signal strength *A*. In the range −17 ≲ *d* ≲ −14, max_τ_ *C* (τ) ≳ 0.8 (indicated by the black region) was obtained in 0.1 ≲ *A* ≲ 1. With increasing value of *d*, the region occupied by *A*, satisfied by max_τ_ *C*(τ) ≳ 0.8, expanded to a smaller *A* in the range −14 ≲ *d* ≲ −12, where the laminar movement became dominant. In particular, the minimum signal strength *A*, satisfied by max_τ_ *C*(τ) ≳ 0.8, attained a value *A* ≈ 10^−3^ at −13 ≲ *d* ≲ *d*_thr_ (≈ −12). With regard to delay ∣*τ*∣, the green filled circles in Fig 11 indicate the points with max_*τ*_ *C*(*τ*) > 0.8 and ∣*τ*∣ < 1.5 [ms]. In the above region (−13 ≲ *d* ≲ *d*_thr_(≈ −12)), these points distributed at the side of *d*_thr_. This region included the points attained promptness (∣*τ*∣ < 1.5 [ms]) in comparison with the other chaotic region, e.g., ∣*τ*∣ ≈ 2.7 [ms] at *d* = −16, where the system exhibited primarily turbulent movement (see Fig 6(d)). Moreover, in the periodic state (*λ*_1_ ≈ 0) region of *d* (*d* > *d*_thr_), max_*τ*_ *C*(*τ*) > 0.8 could not be attained in 1 × 10^−3^ ≲ *A* ≲ 1.0. Thus, signal response in chaotic states was more sensitive than in the periodic state. In particular, the chaotic states along the boundary between the chaotic and periodic states, called the edge of chaos [19, 36], exhibited the highest sensitivity and the promptest response among all chaotic states. Note that the distribution of points satisfied with the promptness was localized to a small boundary region in the region with high sensitivity.

**Fig 12:**
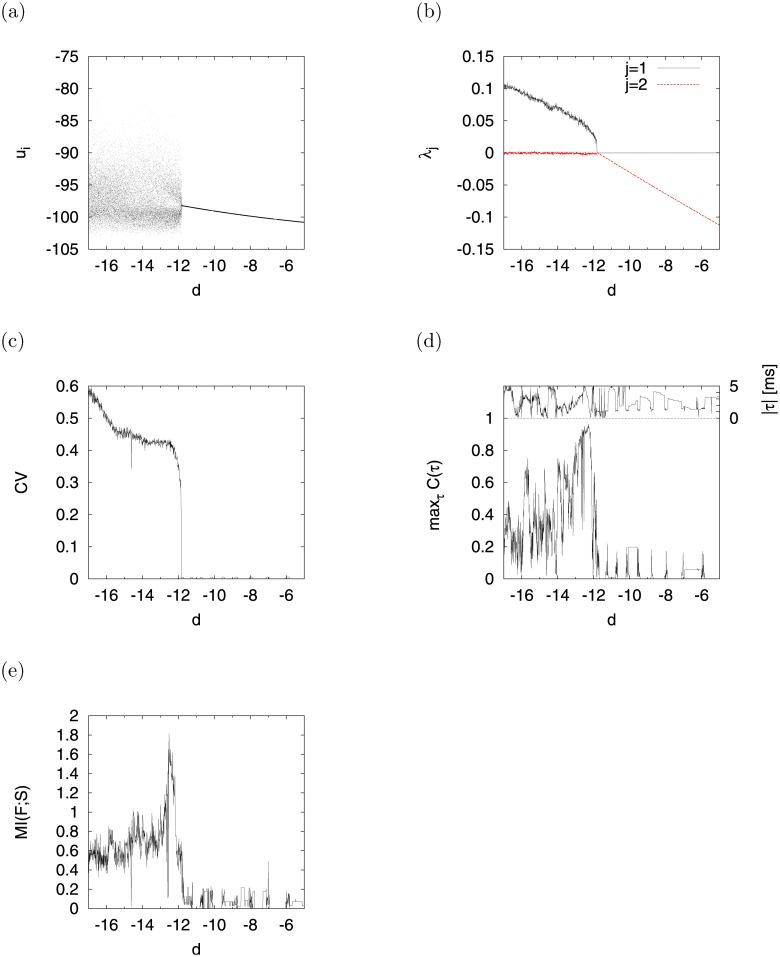
Dependence of bifurcation and signal response on parameter *d* in CR. Under the condition of weaker signals (*A* = 0.01) than those shown in Figs 7 and 10. (a) Bifurcation diagram of *u*_*i*_. (b) *λ*_*j*_. (c) *CV*.(d) max_*τ*_ *C*(*τ*). (Upper part indicates time delay ∣*τ*∣). (e) *MI*(*F*; *S*) (*a* = 0.2, *b* = 2, *c* = −56, *I* = −99, *f*_0_ = 0.1).

**Fig 13:**
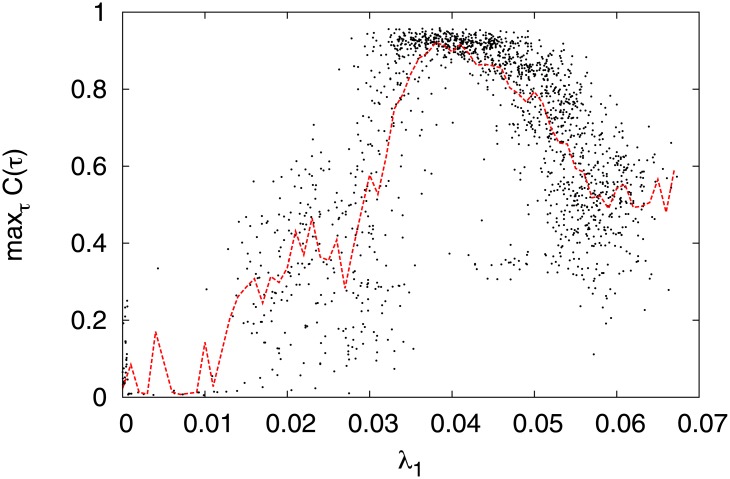
Scatter plot of max_*τ*_ *C*(*τ*) and *λ*_1_ in region −13.5 ≤ *d* ≤ −11 from Fig 12. The red dotted line indicates the mean value of max_*τ*_ *C*(*τ*) in bin *λ*_1_ with window Δ*λ*_1_ = 0.001.

We show the exact dependence between bifurcation and signal response given a weaker signal (*A* = 0.01) than the one used in the section “Dependence on parameter *d*,” which was set at approximately the strength of a signal to be detected almost on the edge of chaos. Fig 12 shows the bifurcation diagrams of *u*_*i*_ ((a)), *λ*_*j*_ (*j* = 1, 2) ((b)), *CV* ((c)), max_*τ*_ *C*(*τ*) ((d)), and *MI*(*F*; *S*) ((e)) as functions of *d*. *u*_*i*_ exhibited chaotic and irregular activity (*λ*_1_ > 0, *CV* ≈ 0.5), and periodic movement with slight motion in its range *u*_*i*_ ≈ 0.1 in the regions −17 ≲ *d* ≲ −12 and −12 ≲ *d* ≲ −5, respectively. The signal response in the region −17 ≲ *d* ≲ −14, which exhibited the impressive performance (max_*τ*_ *C*(*τ*) ≈ 0.9 and *MI*(*F*; *S*) ≈ 1.7) at *A* = 0.3 (see Fig 10), degraded such as at max_*τ*_ *C*(*τ*) ≲ 0.7 and *MI*(*F*; *S*)≲ 1. Nonetheless, the signal response at the edge of chaos maintained satisfactory performance (max_*τ*_ *C*(*τ*) ≈ 0.9 at *d* ≈ −12.19 and *MI*(*F*; *S*) ≈ 1.6 at *d* ≈ −12.5) and rapidness (∣*τ*∣ ≈ 0.1 [ms]). Further, Fig 13 shows the relationship between max_*τ*_ *C*(*τ*) and *λ*_1_ in the region −13.5 ≤ *d* ≤ −11 of Fig 12. The red dotted line indicates the mean value of max_*τ*_ *C*(*τ*) in the bin *λ*_1_ with window Δ*λ*_1_ = 0.001. From this result, we see max_*τ*_ *C*(*τ*) recorded a peak (≈ 1.0) at *λ*_1_ ≈ 0.04, i.e., we confirmed that signal response in CR has an unimodal maximum with respect to the degree of stability for chaotic orbits.

### Dependence on signal frequency *f*_0_

**Fig 14:**
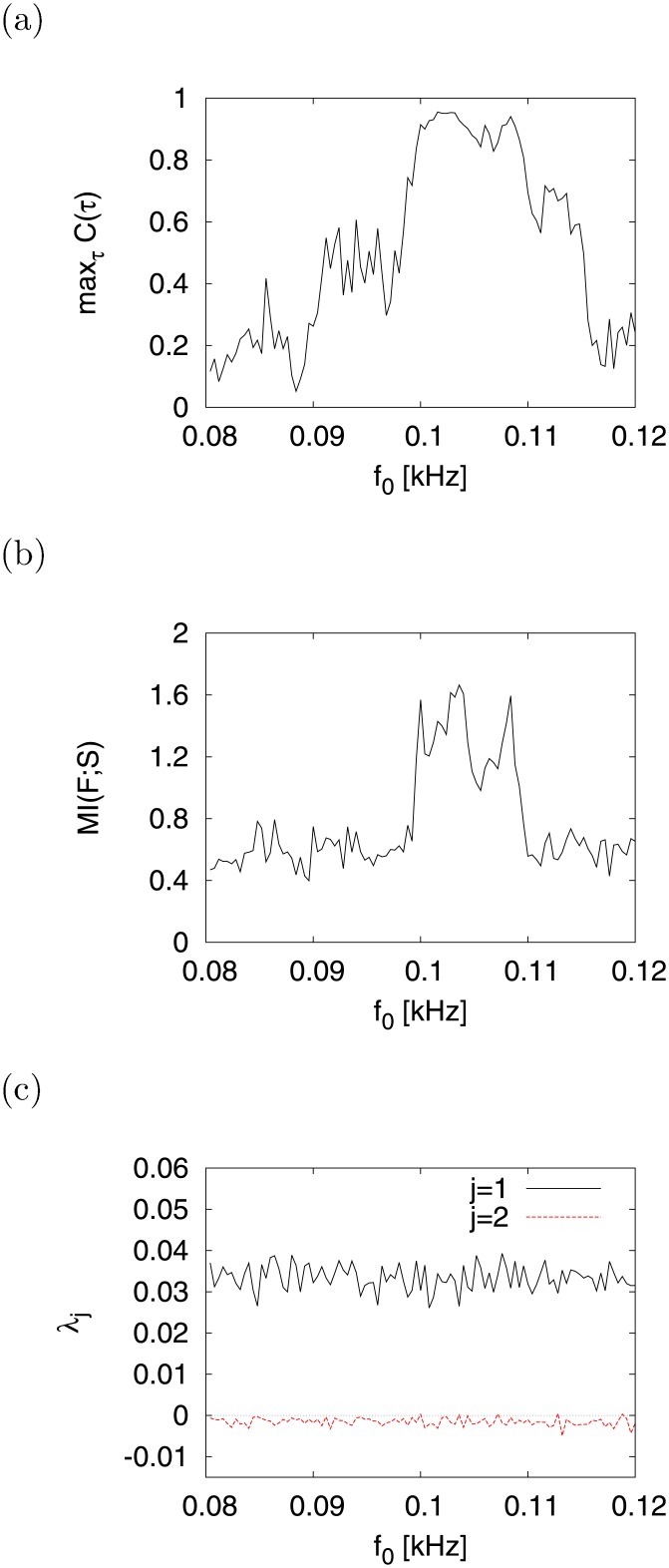
Dependence of signal response on signal frequency *f*_0_. (a) max_*τ*_ *C*(*τ*). (b) *MI*(*F*; *S*). (c) *λ*_*j*_. (*a* = 0.2, *b* = 2, *c* = −56, *I* = −99, *d* = −12.19, *A* = 0.01).

Finally, we evaluated the dependence of signal response on signal frequency in CR under the condition *A* = 0.01, *d* = −12.19. As shown in Fig 14, the dependence of max_*τ*_ *C*(*τ*) ((a)) and *MI*(*F*; *S*) ((b)) on signal frequency *f*_0_ recorded a peak at *f*_0_ ≈ 0.103 [kHz] with chaotic state (*λ*_1_ > 0 ((c))). Thus, CR has a resonance frequency, as is the case with resonance phenomena in general.

## Conclusion

In this paper, we examined the chaotic characteristics of the Izhikevich neuron model in detail by using Lyapunov exponents with a saltation matrix and Poincaré section methods, and discovered two distinctive states: a chaotic state with primarily turbulent movement and an intermittent chaotic state. In order to evaluate the signal response of CR in these classified states, we introduced an extended Izhikevich neuron model by considering a weak periodic signal, and defined a cycle histogram of neuron spikes, and the corresponding mutual correlation and information. Through computer simulations, we confirmed that both chaotic states in CR can sensitively respond to weak signals, and that the intermittent chaotic state exhibited a significantly prompter signal response than the chaotic state with primarily turbulent movement. In particular, the sensitivity and rapidity at the edge of the chaos, located along the border of the intermittency chaos and the periodic state, recorded the highest values of all other chaotic states. Furthermore, we confirmed that signal response in CR is dependent on the frequency of signal input, as is the case with resonance phenomena in general.

From the results obtained here, we expect that the Lyapunov exponent with a saltation matrix can be applied to other reset systems with a state jump, such as control systems and models in the social and financial sciences, as an index to determine whether they are chaotic. With regard to the CR phenomenon, we believe that CR plays an important role in information transmission in the nervous systems. From an engineering viewpoint, the high signal response efficiency in CR can be utilized for the development of devices to detect weak signals.

Another subject of research based suggested by this study is the evaluation of signal response in neuron assemblies consisting of Izhikevich neurons. We are currently exploring the coupling strength dependency of signal response in neuron assemblies.

## Supporting Information

S1 Data Data for Fig 1. (ZIP)

S2 Data Data for Fig 2. (ZIP)

S3 Data Data for Fig 3. (ZIP)

S4 Data Data for Fig 4. (ZIP)

S5 Data Data for Fig 5. (ZIP)

S6 Data Data for Fig 6. (ZIP)

S7 Data Data for Fig 7. (ZIP)

S8 Data Data for Fig 8. (ZIP)

S9 Data Data for Fig 9. (ZIP)

S10 Data Data for Fig 10. (ZIP)

S11 Data Data for Fig 11. (ZIP)

S12 Data Data for Fig 12. (ZIP)

S13 Data Data for Fig 13. (ZIP)

S14 Data Data for Fig 14. (ZIP)
